# Ethyl Acetate Fraction from *Eucommia ulmoides* Ameliorates Particulate Matter (PM)_2.5_-Induced Intestinal Damage by Restoring Barrier Integrity and Regulating Inflammatory Responses

**DOI:** 10.4014/jmb.2504.04002

**Published:** 2025-07-18

**Authors:** Min Ji Kim, Jong Min Kim, Hyo Lim Lee, Ho Jin Heo

**Affiliations:** 1 Division of Applied Life Science (BK21), Institute of Agriculture and Life Science, Gyeongsang National University, Jinju 52828, Republic of Korea; 2 World Institute of Kimchi, Gwangju 61755, Republic of Korea; 3 Korea Food Research Institute, Wanju 55365, Republic of Korea

**Keywords:** *Eucommia ulmoides*, particulate matter, intestinal microenvironment, tight junction, antioxidant system, inflammation

## Abstract

This study investigated the protective effect of ethyl acetate fraction from *Eucommia ulmoides* leaf (EFEL) against intestinal dysfunction induced by chronic exposure to particulate matter (PM)_2.5_ in BALB/c mice. EFEL treatment suppressed reactive oxygen species (ROS) production and cellular death from PM_2.5_-induced HT29 cells. EFEL supplementation ameliorated PM_2.5_-induced intestinal damage by regulating antioxidant biomarkers, including reduced glutathione and superoxide dismutase, malondialdehyde levels, and myeloperoxidase activity. EFEL modulated the gut microbiota composition by increasing beneficial bacteria, such as *Lactobacillus* and *Alistipes*, and reducing the abundance of pathogenic bacteria, such as *Helicobacter* and *Clostridia* UCG-014, thus contributing to the restoration of the intestinal microenvironment. Furthermore, EFEL regulated the expression of tight junction proteins and inflammatory biomarkers in intestinal tissue. These findings suggest that EFEL may serve as a promising functional food material with the potential to alleviate PM_2.5_-induced intestinal barrier dysfunction by regulating oxidative stress, inflammation, and microbiota homeostasis.

## Introduction

Particulate matter (PM) is classified into PM_10_ and PM_2.5_ depending on its diameter [1]. Among them, PM_2.5_ is very small, with a diameter of 2.5 μm or less, and penetrates the body through the respiratory system and causes physiological damage to various tissues [2]. Recent studies have revealed that PM has a harmful effect not only on the lungs but also in the gut tissue, which can affect systemic health by inducing disturbance of the intestinal environment [3]. Although PM_2.5_ is commonly associated with pulmonary toxicity, increasing evidence suggests it can also enter the gastrointestinal tract via unintentional ingestion through swallowing particles trapped in the upper airways, contaminated food or water, or mucosal transfer [4]. These particles can exert direct toxic effects on the intestinal epithelium, leading to barrier dysfunction and local inflammation [2]. In particular, PM_2.5_ changes the composition of the intestinal microbiota, resulting in a decrease in beneficial bacteria and an increase in pathogenic bacteria, which induces dysbiosis of the intestinal microbiota [4]. PM has been shown to reduce microbial diversity, decrease beneficial genera such as *Lactobacillus* and *Bifidobacterium*, and promote the proliferation of pro-inflammatory taxa [5]. These alterations in gut microbiota are closely associated with intestinal barrier dysfunction and the initiation of chronic inflammatory conditions [6]. In addition, PM reduces the expression of tight junction (TJ) proteins, such as occludin and claudin-1, that maintain the bond between intestinal epithelial cells, increase intestinal barrier permeability, and induce an inflammatory response due to external stimuli [7]. Also, Toll-like receptor 4 (TLR4), nuclear factor kappa B (NF-κB), and c-Jun *N*-terminal kinases (JNK) signaling pathways are activated, and the secretion of inflammatory cytokines such as interleukin (IL)-1β and tumor necrosis factor-α (TNF-α) increases, which disrupts the immune homeostasis of intestinal tissue and accelerates tissue damage [8]. Moreover, PM contains various harmful substances, including transition metals, polycyclic aromatic hydrocarbons, and organic compounds, which catalyze excessive production of reactive oxygen species (ROS) in intestinal cells. This imbalance between ROS generation and antioxidant defenses contributes to oxidative stress in the gut microenvironment. These inflammatory and oxidative responses also lead to mitochondrial dysfunction in intestinal epithelial cells and activate apoptotic signaling pathways such as B-cell leukemia/lymphoma 2 (Bcl-2)-associated X (Bax)/Bcl-2 imbalance and caspase activation, contributing to cell loss and further compromising barrier integrity [9]. These mechanisms ultimately cause structural and functional damage to the intestinal lining, leading to systemic inflammation and various metabolic abnormalities [10]. Therefore, the intake of bioactive substances with antioxidant and anti-inflammatory properties is attracting attention as a strategy to alleviate intestinal tissue damage caused by PM_2.5_, and in particular, natural substances can work effectively to maintain intestinal health through various mechanisms such as improving intestinal microflora, protecting the intestinal barrier, suppressing inflammation, and reducing apoptosis [11].

*Eucommia ulmoides* is a traditional medicinal plant used in East Asia and has been widely used since ancient times for its effects such as tonic, antihypertensive, and anti-aging [12]. In particular, *Eucommia* leaves contain various physiologically active compounds such as chlorogenic acid, lignan, iridoid, and flavonoid, and these are known to have various physiologically active functions such as antioxidant, anti-inflammation, neuroprotection, and immunomodulation [13,14]. Recently, research has been conducted on the positive effects of *Eucommia* on gastrointestinal health [15,16]. However, the effects of *Eucommia* on improving the intestinal environment and tissue damage induced by PM_2.5_ have not yet been sufficiently elucidated. In particular, there is insufficient scientific evidence on the effects of *Eucommia* on the recovery of the intestinal tissue environment disturbed by PM_2.5_, such as regulation of intestinal microflora, protection of TJ proteins, and inhibition of inflammatory responses. This study aimed to comprehensively evaluate the intestinal tissue protective effects of ethyl acetate fraction from *E. ulmoides* leaf (EFEL) in a mouse model of intestinal damage induced by PM_2.5_ exposure. By clarifying whether EFEL can improve intestinal health, such as restoring intestinal microflora balance, maintaining intestinal barrier function, and suppressing inflammatory responses, we aimed to suggest using *Eucommia* as a functional food material.

## Materials and Methods

### Sample Preparation

*E. ulmoides* leaves were sourced from Yeongcheon, Republic of Korea, in April 2019 and authenticated by the National Institute of Forest Science (Republic of Korea). Dried leaves were extracted in 40% ethanol for 2 h at 40°C, and the resulting solution was fractionated sequentially with n-hexane, chloroform, and ethyl acetate. Each fraction was freeze-dried and stored at −70°C. Based on previous findings indicating significant antioxidant activity among the fractions, the ethyl acetate fraction (EFEL) was selected for the experiments. The major compounds of EFEL, such as chlorogenic acid, rutin, and quercetin derivatives, have been previously identified [14].

### Cell Culture and Treatment

HT29 human intestinal epithelial cells were obtained from the Korean Cell Line Bank (Republic of Korea) and maintained in DMEM supplemented with 10% fetal bovine serum, 50 U/ml penicillin, and 100 μg/ml streptomycin at 37°C under a humidified atmosphere containing 5% CO_2_.

To evaluate cytotoxicity, HT29 cells were treated with EFEL at 10, 20, or 50 μg/ml concentrations and 200 μM vitamin C. After 3 h, 100 μg/ml of PM_2.5_ was added and incubated for 24 h. Cell viability was assessed using the MTT assay, with 5 mg/ml MTT solution applied for 3 h. Formazan formation was quantified by measuring absorbance at 570 nm with a reference at 690 nm.

For intracellular ROS analysis, cells were similarly treated with EFEL and PM_2.5_, followed by incubation with 5 mg/ml of dichlorodihydrofluorescein diacetate (DCFH-DA) for 40 min. Fluorescence intensity was detected using excitation at 485 nm and emission at 535 nm.

### Animals and Diet

Six-week-old male BALB/c mice were purchased from Samtako (Republic of Korea). Mice were housed in a controlled environment (22 ± 2°C, 55% humidity, 12-h light/dark cycle), group 3–4 per cage. Animals were divided into four groups: (1) normal control (NC; vehicle intake with clean air), (2) PM_2.5_-exposed without treatment, and (3) and (4) PM_2.5_-exposed groups receiving EFEL at 20 or 40 mg/kg body weight (EFEL20 or EFEL40, respectively). EFEL was administered orally for 12 weeks. PM_2.5_, obtained from Power Technology Inc.,(Arizona Test Dust, USA), was aerosolized and introduced into exposure chambers for 5 h daily during the treatment period. Elemental analysis of the PM_2.5_ used in this study has been previously reported [17], revealing the presence of Fe (16.91 mg/g), Mg (6.26 mg/g), Mn (0.58 mg/g), Ba (0.22 mg/g), Zn (0.07 mg/g), Cu (0.05 mg/g), Pb (0.03 mg/g), Li (0.02 mg/g), and Cr (0.01 mg/g). At the end of the exposure period, animals were euthanized using CO_2_ anesthesia, and blood and intestinal samples were collected. All procedures were approved by the Institutional Animal Care and Use Committee of Gyeongsang National University (Approval No. GNU-200302-M0007; Date: March 2, 2020).

### Tissue Preparation

Collected intestinal tissues were homogenized at 4°C using a bullet blender (Next Advance Inc., USA) in phosphate-buffered saline (PBS) or phosphate buffer containing 1 mM EDTA (pH 6.7). Whole blood was centrifuged at 13,000 ×*g* for 10 min, and the resulting serum was used for further analyses.

### Antioxidant Biomarkers

Lipid peroxidation was assessed by determining malondialdehyde (MDA) levels. Tissue homogenates were reacted with 1% phosphoric acid and 0.67% thiobarbituric acid at 95°C for 1 h, and absorbance was read at 532 nm [17].

Reduced glutathione (GSH) levels were determined using a fluorescence-based assay. Supernatants of homogenized tissue treated with 5% metaphosphoric acid were reacted with o-phthaldialdehyde in Tris-HCl buffer and NaOH. Fluorescence was measured at 320 nm excitation and 420 nm emission using a Tecan Infinite 200 reader [17].

Superoxide dismutase (SOD) activity was evaluated using a commercial kit (Dojindo, Japan) according to the manufacturer's instructions. Tissues were processed in PBS, and the resulting supernatants were used for analysis.

Myeloperoxidase (MPO) activity was assessed in homogenates prepared with hexadecyltrimethylammonium bromide in phosphate buffer. After centrifugation, supernatants were incubated with o-dianisidine dihydrochloride and hydrogen peroxide, and absorbance was measured at 450 nm [17].

Serum lactate dehydrogenase (LDH) levels were measured using a Fuji Dri-Chem 4000i analyzer (Fujifilm, Japan).

### Short Chain Fatty Acid (SCFA) in Feces Contents

Fecal samples were homogenized in 5 mM NaOH, centrifuged, and the supernatant derivatized with propanol, pyridine, and propyl chloroformate. After hexane extraction and additional centrifugation, SCFA levels were analyzed using a gas chromatograph (Agilent 7890A) following the method described by Kim *et al*. [17].

### 16s rRNA Gene Amplification

Following genomic DNA extraction, the 16S rRNA gene amplified targeting the V3–V4 hypervariable regions was immediately conducted. The PCR was performed using the KAPA HiFi HotStart ReadyMix (2×) (Roche, Switzerland) to ensure high-fidelity amplification. Universal primers specific for the bacterial 16S rRNA gene, integrated with Illumina overhang adapter sequences, were employed: forward primer 341F (5’-TCGTCG GCAGCGTCAGATGTGTATAAGAGACAGCCTACGGGNGGCWGCAG-3’) and reverse primer 806R (5’-GTCTCGTGGGCTCGGAGATGTGTATAAGAGACAGGACTACHVGGGTATCTAATCC-3’) (Illumina, USA).

### Library Preparation, Quantification, and Sequencing

To remove residual primers and primer-dimer artifacts from the PCR products, purification was performed using AMPure XP magnetic beads (Beckman Coulter, Cat. No. A63881, USA). Subsequent dual-index barcoding and the addition of Illumina sequencing adapters were performed using the Nextera XT Index Kit (Illumina, USA). A second round of purification using AMPure XP beads was conducted to ensure library quality. Before sequencing, the concentration of each library was quantified using a Qubit 3.0 fluorometer (Thermo Fisher Scientific, USA), and the fragment size distribution and integrity were assessed using a 2100 Bioanalyzer system (Agilent Technologies, USA). Sequencing was performed on the Illumina MiSeq platform (Illumina) according to the manufacturer’s instructions.

### Western Blot Analysis

Intestinal tissues were lysed in a buffer containing protease inhibitors (GeneAll Biotechnology, Republic of Korea). Protein extracts were clarified by centrifugation (13,000 ×*g*, 10 min, 4°C), resolved by SDS-PAGE, and transferred to membranes. Blots were incubated with primary antibodies overnight at 4°C, followed by secondary antibodies at 25°C for 1 h. Detection was performed using an iBright Imager (Thermo Fisher Scientific), and band intensities were quantified using ImageJ software (NIH, USA).

### Statistical Analysis

Experimental results are presented as the mean ± standard deviation (SD). Comparisons between groups were performed using one-way analysis of variance, followed by Tukey’s post-hoc analysis (GraphPad software ver. 10.0; Prism Inc., USA). Statistical significance was analyzed as *P* < 0.05, 0.01, and 0.001. Pearson’s correlation was conducted using RStudio (Ver. 4.2.2, RStudio, Inc., USA) and visualized as a heat map.

## Results

### Cellular Protective Effect

**Fig. 1:**
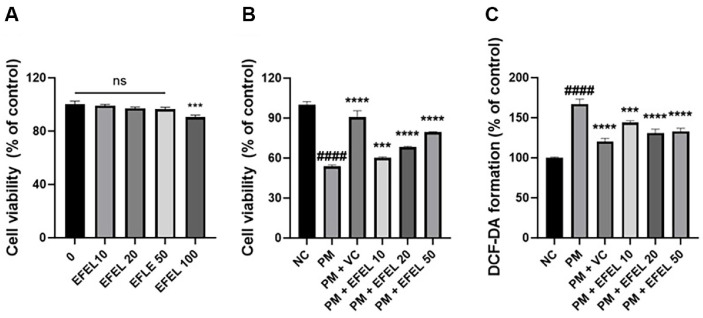
Protective effect of ethyl acetate fraction from *Eucommia ulmoides* (EFEL). Cell viability without PM_2.5_ (**A**) and with PM_2.5_ (**B**). Intracellular ROS production (**C**) on HT29 cells. Results shown are mean ± SD (*n* = 5). Data were considered statistically significant at *p* < 0.05 and different small letters represent statistical differences. ^####^*P* < 0.0001 compared to the NC group. *P < 0.05, **P < 0.01, ***P < 0.001, ****P < 0.0001 compared to the PM group. ns indicates no significant difference between groups. Vitamin C (200 μM) was used as a positive control.

The cytotoxicity of EFEL was evaluated and no effect was observed up to 50 μg/ml (Fig. 1A). The cell viability of the PM_2.5_-induced group (53.79%) was reduced compared to the normal control group (100%) (Fig. 1B). However, that of the vitamin C and EFEL-treated groups was increased (vitamin C, 90.77%; 10 μg/ml, 60.19%; 20 μg/ml, 68.28%; 50 μg/ml, 79.32%, respectively) compared to the PM_2.5_-induced group. The ROS contents of the PM_2.5_-induced group (167.10%) were increased compared to the normal control group (100%) (Fig. 1C). However, that of the vitamin C and EFEL-treated groups decreased (vitamin C, 120.33%; 10 μg/ml, 143.89%; 20 μg/ml, 131.11%; 50 μg/ml, 133.17%, respectively) compared to the PM_2.5_-induced group.

### Colon Length

**Fig. 2:**
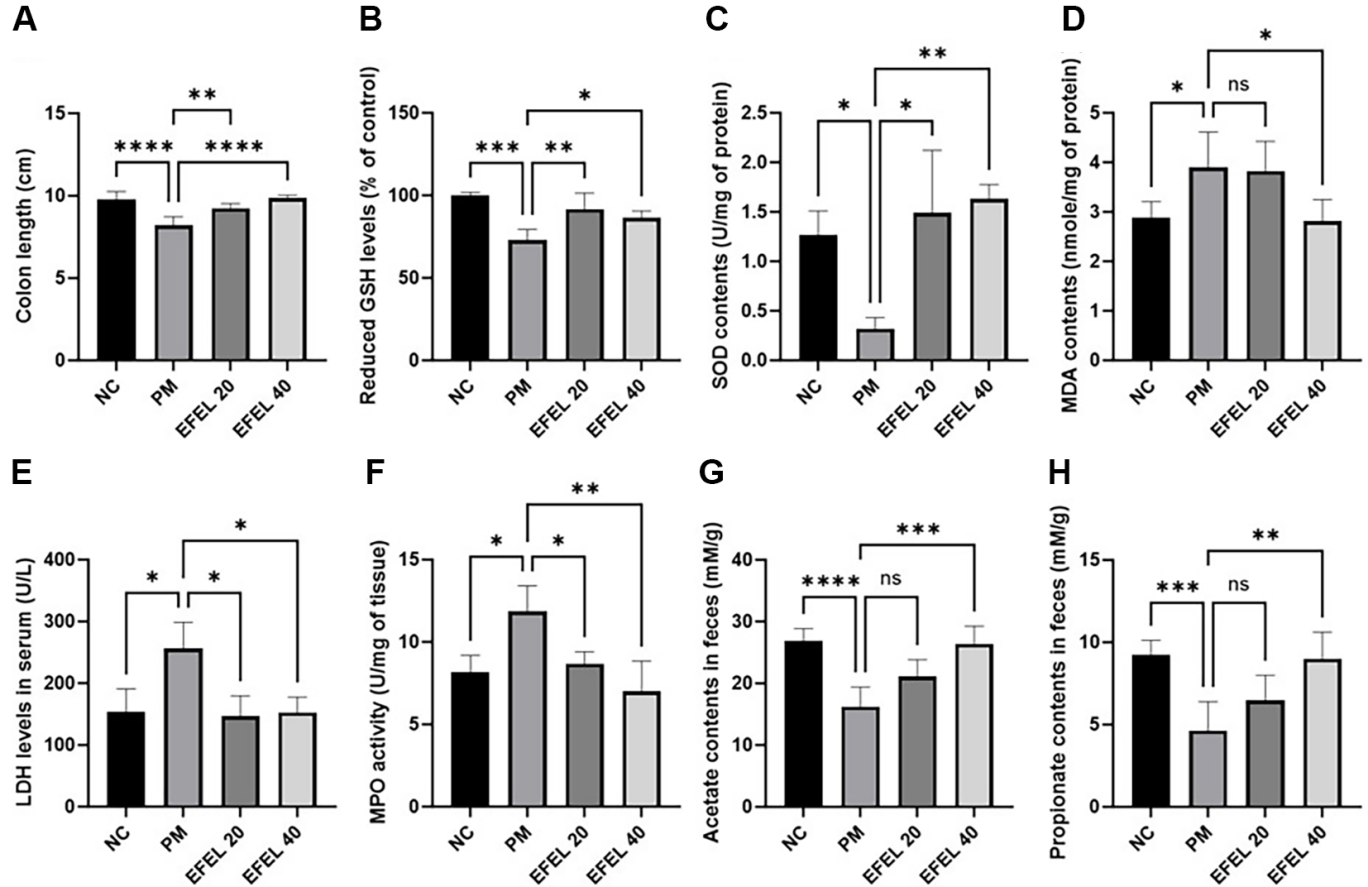
Protective effect of ethyl acetate fraction from *Eucommia ulmoides* (EFEL) on colon length (**A**), reduced GSH content (**B**), SOD content (**C**), MDA content (**D**), LDH level (**E**), MPO activity (**F**) fecal acetate content (**G**), and fecal propionate content (**H**) in intestinal tissue. Results shown are mean ± SD (*n* = 5). Data were considered statistically significant at *p* < 0.05 and different small letters represent statistical differences. *P < 0.05, **P < 0.01, ***P < 0.001, ****P < 0.0001 between each group. ns indicates no significant difference between groups. EFEL was administered orally at 20 (EFEL 20) and 40 (EFEL 40) mg/kg body weight.

Colon length is shown in Fig. 2A. The colon length of the PM group (8.20 cm) decreased more than the that of the NC group (9.78 cm). While, that of the EFEL group (EFEL20, 9.22 cm; EFEL40, 9.84 cm) was improved more than the PM group.

### Antioxidant Biomarkers

Reduced intestinal GSH levels and SOD activity are shown in Fig. 2B and 2C. The reduced GSH level and SOD activity of the PM group (72.97% and 0.32 U/mg of protein, respectively) was reduced more than that of the NC group (100.00% and 1.27 U/mg of protein, respectively). However, that of the EFEL group (EFEL20, 91.56% and 1.49 U/mg of protein; EFEL40, 86.27% and 1.63 U/mg of protein) improved more than the PM group.

Intestinal MDA content, LDH level, and MPO activity are shown in Fig. 2D-2F. The MDA content, LDH level, and MPO activity of the PM group (3.90 nmole/mg of protein, 256.00 U/L, and 11.86 U/mg, respectively) decreased more than the that of the NC group (2.89 nmole/mg of protein, 154.00 U/L, and 8.14 U/mg, respectively). However, that of the EFEL group (EFEL20, 3.82 nmole/mg of protein, 146.33 U/L, and 8.67 U/mg; EFEL40, 2.81 nmole/mg of protein, 152.67 U/L, and 6.99 U/mg) improved more than the PM group.

### SCFA Contents in Feces

Fecal SCFAs concentrations are shown in Fig. 2G and 2H. The SCFAs concentration of the PM group (acetate, 16.26 mM/g; propionate, 4.63 mM/g) was downregulated compared to the NC group (acetate, 26.91 mM/g; propionate, 9.23 mM/g). The EFEL 20 group (acetate, 21.09 mM/g; propionate, 6.47 mM/g) and EFEL40 group (acetate, 26.34 mM/g; propionate, 8.99 mM/g) improved the fecal SCFAs concentration.

### Gut Microbiome

**Fig. 3:**
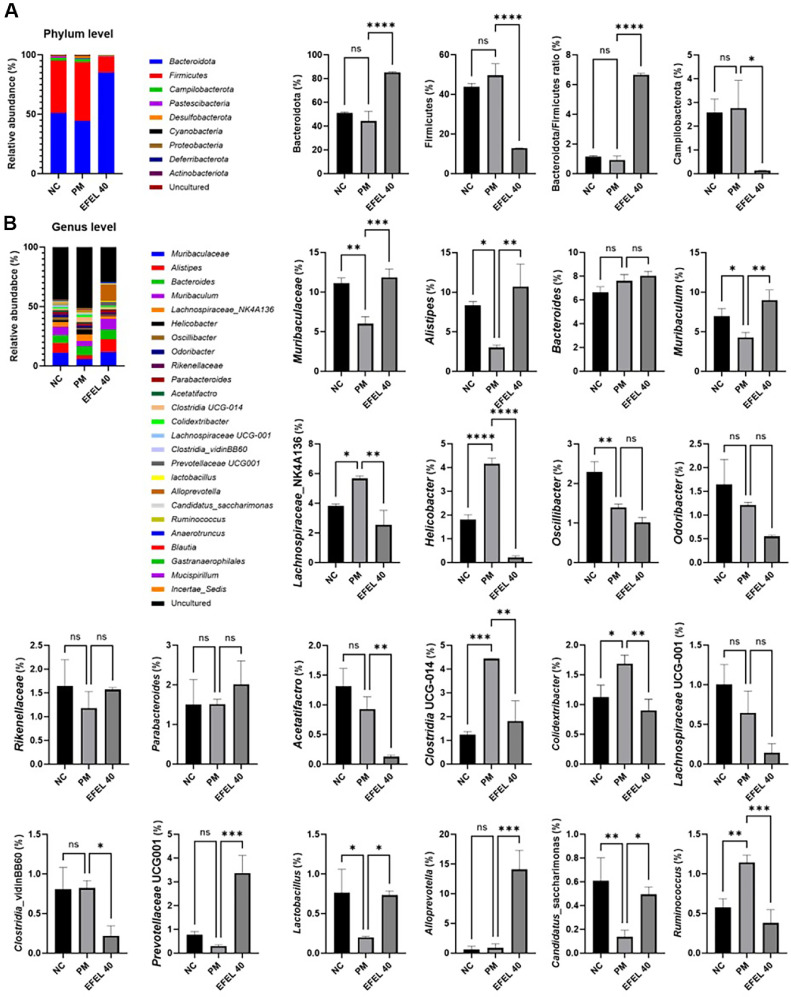
Relative abundance (%) of the gut microbiome at the genera level by ethyl acetate fraction from *Eucommia ulmoides* (EFEL) in PM_2.5_-induced gut dysbiosis. Relative abundances of the Phylum (**A**) and Genus (**B**) in each group. Results shown are mean ± SD (*n* = 3). Data were considered statistically significant at *p* < 0.05 and different small letters represent statistical differences. *P < 0.05, **P < 0.01, ***P < 0.001, ****P < 0.0001 between each group. ns indicates no significant difference between groups. EFEL was administered orally at 40 (EFEL 40) mg/kg body weight.

Gut microbiota changes are presented in Fig. 3. The relative abundances of the phylum of the NC group (Bacteroidetes, 51.14%; Firmicutes, 43.91%; Bacteroidetes/Firmicutes ratio, 1.17; Campilobacterota, 2.58%) and PM group (Bacteroidetes, 44.34%; Firmicutes, 49.58%; Bacteroidetes/Firmicutes ratio, 0.92; Campilobacterota, 2.77%) confirmed no significant differences (Fig. 2A), whereas the relative abundances of the phylum of the EFEL40 group (Bacteroidetes, 85.30%; Firmicutes, 12.82%; Bacteroidetes/Firmicutes ratio, 6.66; Campilobacterota, 0.14%) were significantly changed. The relative abundances of the genus are presented in Fig. 2B. The relative abundances of the genus of the PM group (*Muribaculaceae*, 5.99%; *Alistipes*, 3.00%; *Muribaculum*, 4.28%; *Lachnospiraceae*_NK4A136, 5.67%; *Helicobacter*, 4.16%; *Oscillibacter*, 1.39%; *Acetatifactro*, 0.92%; *Clostridia* UCG-014, 4.43%; *Colidextribacter*, 1.69%; *Clostridia*_vidinBB60, 0.82%; *Prevotellaceae* UCG001, 0.29%; *Lactobacillus*, 0.20%; *Alloprevotella*, 0.86%; *Candidatus*_saccharimonas, 0.14%; *Ruminococcus*, 1.14%; *Anaerotruncus*, 0.27%; *Blautia*, 0.13%; *Gastranaerophilales*, 0.40%; *Mucispirillum*, 0.17%; and *Incertae*_Sedis, 0.50%) were changed compared to NC group (*Muribaculaceae*, 11.10%; *Alistipes*, 8.34%; *Muribaculum*, 6.97%; *Lachnospiraceae*_NK4A136, 3.84%; *Helicobacter*, 1.18%; *Oscillibacter*, 2.29%; *Acetatifactro*, 1.31%; *Clostridia* UCG-014, 1.23%; *Colidextribacter*, 1.13%; *Clostridia*_vidinBB60, 0.81%; *Prevotellaceae* UCG001, 0.77%; *Lactobacillus*, 0.76%; *Alloprevotella*, 0.61%; *Candidatus*_saccharimonas, 0.61%; *Ruminococcus*, 0.57%; *Anaerotruncus*, 0.47%; *Blautia*, 0.31%; *Gastranaerophilales*, 0.25%; *Mucispirillum*, 0.22%; and *Incertae*_Sedis, 0.17%). However, the relative abundances of the genus of the EFEL40 group (*Muribaculaceae*, 11.83%; *Alistipes*, 10.72%; *Muribaculum*, 9.01%; *Lachnospiraceae*_NK4A136, 2.53%; *Helicobacter*, 0.21%; *Oscillibacter*, 1.02%; *Acetatifactro*, 0.13%; *Clostridia* UCG-014, 1.81%; *Colidextribacter*, 0.90%; *Clostridia*_vidinBB60, 0.22%; *Prevotellaceae* UCG001, 3.37%; *Lactobacillus*, 0.73%; *Alloprevotella*, 14.10%; *Candidatus*_saccharimonas, 0.49%; *Ruminococcus*, 0.38%; *Anaerotruncus*, 0.51%; *Blautia*, 0.22%; *Gastranaerophilales*, 0.12%; *Mucispirillum*, 0.01%; and *Incertae*_Sedis, 0.07%) were ameliorated compared to PM group. *Bacteroides*, *Odoribacter*, *Rikenellaceae*, *Parabacteroides*, and *Lachnospiraceae* UCG-001 were no significant differences between all groups.

### Intestinal Protein Expression level

**Fig. 4:**
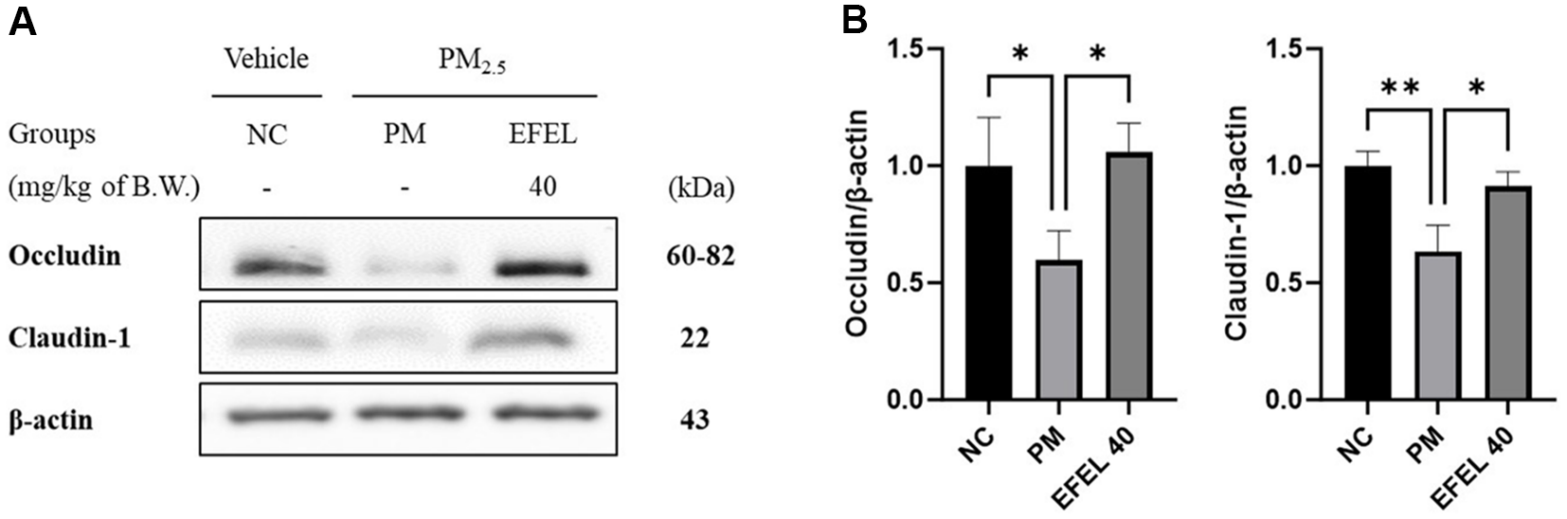
Effect of ethyl acetate fraction from *Eucommia ulmoides* (EFEL) on tight junction protein expression. Western blot band images (**A**). Protein expression levels of Occludin and Claudin-1 (**B**) in intestinal tissues. Results shown are mean ± SD (*n* = 3). Data were considered statistically significant at *p* < 0.05 and different small letters represent statistical differences. *P < 0.05, **P < 0.01, ***P < 0.001, ****P < 0.0001 between each group. ns indicates no significant difference between groups. EFEL was administered orally at 40 (EFEL 40) mg/kg body weight.

Intestinal protein expressions related to TJ are presented in Fig. 4. Occludin (63.30%) and Claudin-1 (59.66%) expression levels of the PM group were reduced compared to the NC group. The EFEL40 group statistically improved Occludin (105.85%) and Claudin-1 (91.21%), expression levels.

**Fig. 5:**
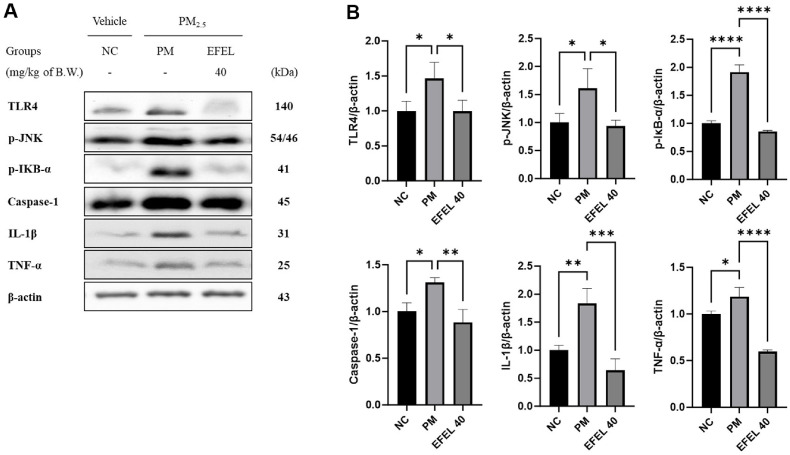
Effect of ethyl acetate fraction from *Eucommia ulmoides* (EFEL) on inflammatory protein expression. Western blot band images (**A**). Protein expression levels of TLR4, p-JNK, p-IκB-α, caspase-1, IL-1β, and TNF-α (**B**) in intestinal tissues. Results shown are mean ± SD (*n* = 3). Data were considered statistically significant at *p* < 0.05 and different small letters represent statistical differences. *P < 0.05, **P < 0.01, ***P < 0.001, ****P < 0.0001 between each group. ns indicates no significant difference between groups. EFEL was administered orally at 40 (EFEL 40) mg/kg body weight.

Intestinal protein expressions related to inflammation are presented in Fig. 5. TLR4 (146.45%), phosphorylated JNK (p-JNK) (161.10%), phosphorylated NFKB inhibitor α (p-IκB-α) (191.26%), caspase-1 (131.16%), IL-1β (183.31%), and TNF-α (118.48%) expression levels of the PM group were increased compared to the NC group. However, the EFEL40 group statistically down-regulated TLR4 (99.62%), p-JNK (93.59%), p-IκB-α (85.39%), caspase-1 (88.49%), IL-1β (64.27%), and TNF-α (59.57%) levels.

**Fig. 6:**
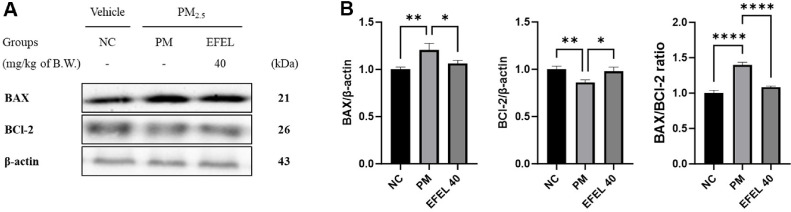
Effect of ethyl acetate fraction from *Eucommia ulmoides* (EFEL) on apoptotic protein expression. Western blot band images (**A**). Protein expression levels of BAX, BCl-2, and BAX/BCl-2 ratio (**B**) in intestinal tissues. Results shown are mean ± SD (*n* = 3). Data were considered statistically significant at *p* < 0.05 and different small letters represent statistical differences. *P < 0.05, **P < 0.01, ***P < 0.001, ****P < 0.0001 between each group. ns indicates no significant difference between groups. EFEL was administered orally at 40 (EFEL 40) mg/kg body weight.

Intestinal protein expressions related to apoptosis are presented in Fig. 6. BAX (120.45%) expression levels and BAX/BCl-2 ratio (1.40) of the PM group increased compared to the NC group. However, the EFEL40 group statistically down-regulated BAX (160.17%) level and BAX/BCl-2 ratio (1.08). BCl-2 (86.15%) expression levels of the PM group were reduced compared to the NC group. However, the EFEL40 group statistically up-regulated BCl-2 (97.97%) levels.

### Correlation Analysis

**Fig. 7:**
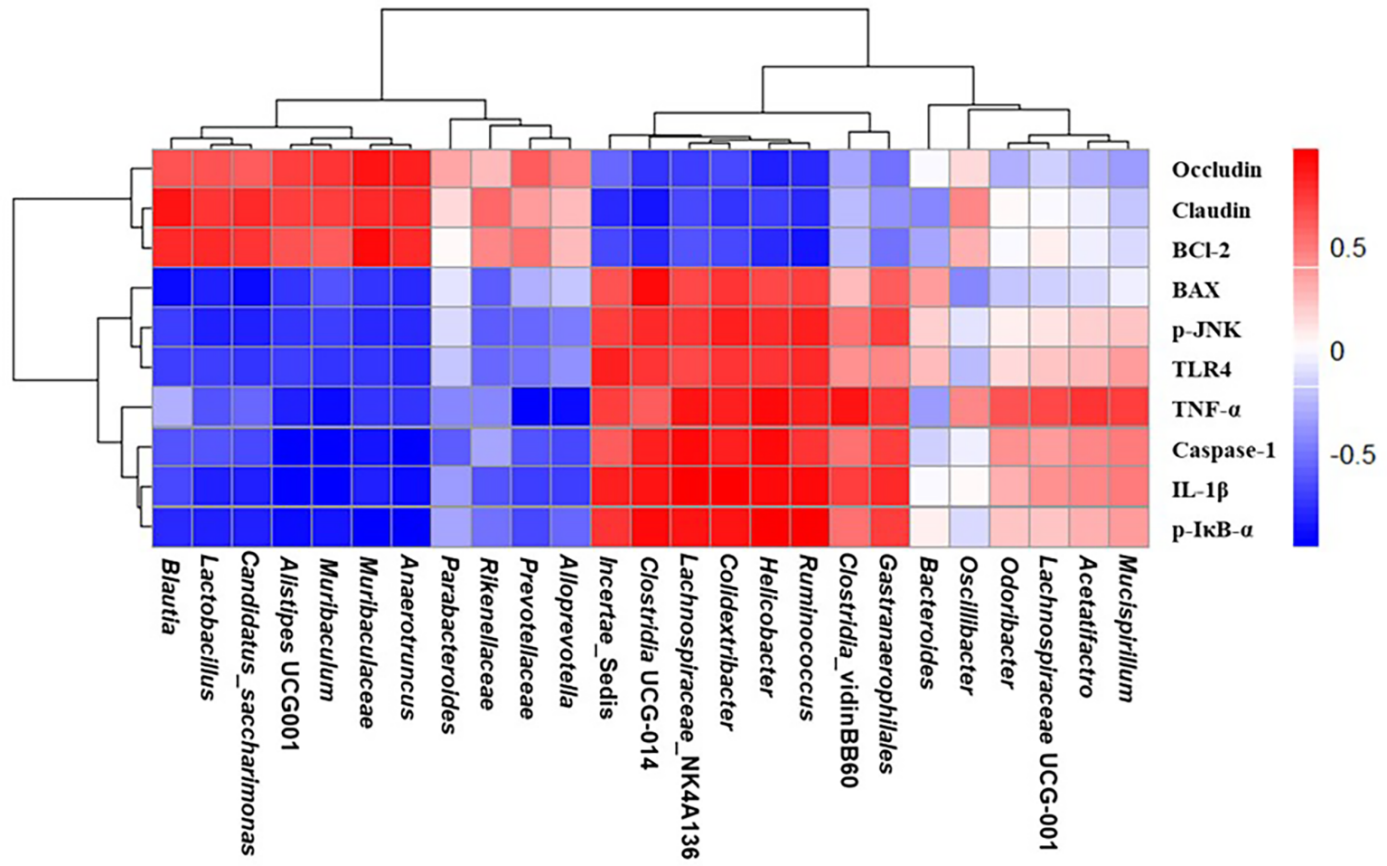
Effect of ethyl acetate fraction from *Eucommia ulmoides* (EFEL) on correlation analysis. Heat map of correlation analysis between gut microbiota and biomarkers analyzed in intestinal tissues.

Correlation analysis between intestinal gene expression markers and microbiota composition is presented in Fig. 7. This analysis included tight junction proteins, including Occludin and Claudin-1, inflammatory mediators, including IL-1β, TNF-α, TLR4, p-JNK, p-IκBα, and Caspase-1, and apoptosis-related factors, including BAX and Bcl-2, as well as representative gut microbial taxa altered by PM_2.5_ exposure and EFEL treatment. Especially pro-inflammatory markers such as TLR4, p-JNK, IL-1β, and TNF-α showed positive correlations with *Helicobacter*, *Clostridia* UCG-014, and *Colidextribacter*, which were enriched in the PM group. In contrast, beneficial genera such as *Muribaculaceae*, *Alistipes*, *Alloprevotella*, and *Lactobacillus* were positively correlated with anti-inflammatory markers (Bcl-2) and barrier-related proteins such as Occludin and Claudin-1. EFEL treatment appeared to reverse many of these PM-induced correlations, suggesting a potential role in restoring intestinal immune balance and microbiota homeostasis. These results imply that the microbial shifts induced by PM2.5 are closely linked to host inflammatory, apoptotic, and barrier-related gene expression and that EFEL modulates these interactions through its regulatory effects on gut microbiota and host signaling.

## Discussion

In this study, we evaluated the protective effect of EFEL on intestinal tissue damage induced by PM_2.5_ exposure. PM_2.5_ causes structural and functional damage to intestinal tissue through complex mechanisms, such as an imbalance of intestinal microbiota, decreased expression of TJ proteins, and induction of inflammatory responses [18]. The results of this study show that EFEL effectively suppresses PM_2.5_-induced intestinal damage and contributes to maintaining homeostasis of the intestinal environment with antioxidant and anti-inflammatory effects and improvement of intestinal microbiota.

PM_2.5_ is composed of various heavy metals, including lead, cadmium, and nickel, polycyclic aromatic hydrocarbons, and volatile organic compounds (VOCs) [19]. It is a significant environmental toxic factor that induces strong oxidative stress when inhaled into the body [20]. In particular, PM_2.5_ can be directly inhaled into the intestines or indirectly reach intestinal tissues through the circulatory system, causing excessive production of ROS in intestinal epithelial cells and resulting in the collapse of the antioxidant defense system [21]. At this time, ROS accumulation accelerates the damage to cell structures and biomolecules by inducing lipid peroxide accumulation along with an imbalance in the antioxidant enzyme system, such as GSH depletion and decreased SOD/catalase activity [22]. In this study, we confirmed that the antioxidant system in the intestinal tissue was seriously damaged by the significant decrease in GSH, significant increase in MDA, and decreased SOD activity in the PM exposure group. Damage to the antioxidant system goes beyond simple ROS accumulation [19]. It causes oxidative modification of lipids, proteins, and DNA in intestinal epithelial cells, which leads to the collapse of the cell membrane structure and metabolic function [23]. In particular, oxidative damage to the intestinal mucosa is accompanied by inhibition of the expression of TJ proteins, occludin and claudin-1, which weakens the bond between intestinal cells and causes intestinal barrier dysfunction [24]. This barrier damage ultimately leads to leaky gut syndrome, which increases the penetration of external toxic substances, microorganisms, and endotoxin into the body and can induce a systemic inflammatory response that spreads from local inflammation to systemic inflammation [25]. The destruction of the intestinal antioxidant system is increasingly linked to systemic metabolic disorders, autoimmune diseases, cardiovascular diseases, and neurological inflammation, and the importance of antioxidant strategies to maintain intestinal health is being highlighted [26]. EFEL showed a strong protective effect against such damage. EFEL contains physiologically active ingredients such as 5-*O*-caffeoylquinic acid, quercetin, rutin, and luteolin derivatives, which contribute to ROS removal, increased antioxidant enzyme activity, and inhibition of lipid peroxidation [13]. In this study, the recovery of GSH levels, increased SOD activity, and decreased MDA were confirmed in the EFEL-treated group, suggesting that the antioxidant system was restored. In addition, EFEL effectively suppressed intestinal mucosal damage caused by oxidative stress by contributing to the suppression of intestinal inflammatory responses and the restoration of TJ protein expression.

When PM enters the intestine, physical stimulation and biochemical changes in the intestinal mucosal environment are simultaneously induced [27]. In particular, metal ions, organic compounds, and toxic substances contained in PM cause rapid changes in the intestinal metabolic environment, which are accompanied by abnormalities in various metabolites [28]. These metabolite changes increase oxidative stress in intestinal epithelial cells and affect intestinal pH, mucus secretion, and SCFA production, such as acetate and propionate, thereby changing the microbial habitat environment [29]. In particular, fecal SCFA levels, such as acetate and propionate, were significantly reduced following PM_2.5_ exposure, suggesting the dysbiosis-induced impairment of microbial metabolic activity [30]. As a result, the growth of beneficial bacteria is suppressed, and the proliferation of pathogenic microorganisms is promoted, resulting in dysbiosis of the intestinal microbiota [31]. In this study, an evident microbiota disturbance phenomenon was observed in which the relative abundance of strains such as *Firmicutes* and *Campilobacterota* increased and beneficial bacteria such as *Bacteroidota*, *Lactobacillus*, and *Alistipes* decreased due to exposure to PM_2.5_. As previously reported, this suggests that changes in the intestinal microenvironment lead to the dominance of specific pathogenic bacteria and aggravate intestinal inflammatory responses. In particular, the increase in pathogenic bacteria such as *Helicobacter* can induce intestinal inflammatory responses and weaken the intestinal barrier function, which can lead to the destruction of the homeostasis of the intestinal-immune system [32]. On the other hand, the administration of EFEL significantly improved these PM_2.5_-induced intestinal microbiota changes. EFEL contributed to normalizing the dysbiosis state by promoting the growth of beneficial bacteria and inhibiting the proliferation of pathogenic bacteria. In particular, the recovery of the *Bacteroidota*/*Firmicutes* ratio, the increase in beneficial bacteria (*Alistipes*, *Muribaculaceae*, *Lactobacillus*, etc.), and the decrease in pathogenic strains such as *Helicobacter* and *Clostridia* UCG-014 are interpreted as results that support the ability of EFEL to regulate the intestinal environment. This effect seems to be due to the antioxidant and anti-inflammatory properties of the polyphenols, lignans, and chlorogenic acid contained in EFEL, which improve the intestinal metabolic environment and affect microbial growth conditions [14, 33]. The phenolic and flavonoid compounds in EFEL positively influence the regulation of intestinal microflora. Notably, 5-O-caffeoylquinic acid and quercetin derivatives help stabilize the intestinal metabolic environment by promoting the growth of beneficial bacteria and increasing SCFA production [34]. In the present study, EFEL significantly restored the levels of fecal acetate and propionate, which had been reduced by PM_2.5_ exposure, indicating that EFEL improves microbial metabolic function and contributes to the recovery of gut homeostasis. Furthermore, rutin and luteolin derivatives can effectively address dysbiosis by inhibiting the growth of intestinal inflammatory microflora and helping to restore the *Bacteroidota*/*Firmicutes* ratio [35, 36]. Therefore, this study provides scientific evidence that EFEL can contribute to maintaining intestinal health by effectively regulating changes in intestinal metabolites and resulting microbiota disturbances caused by PM.

PM_2.5_ exposure changes the composition of the intestinal microbiota to a pathogenic status and directly affects the protein expression and immune response of intestinal epithelial cells [37]. As observed in this study, pathogenic strains such as *Helicobacter*, *Clostridia* UCG-014, and *Colidextribacter* became dominant when exposed to PM [38]. In contrast, beneficial bacteria such as *Lactobacillus*, *Alistipes*, *Muribaculum*, and *Ruminococcus* were significantly reduced [39]. This dysbiosis is not limited to a simple change in the microbiota but also changes the intestinal metabolic environment to an inflammatory one, and induces structural changes in the function of epithelial cells [40]. In particular, toxic molecules such as lipopolysaccharides secreted by pathogenic microorganisms activate TLR4 of intestinal epithelial cells and stimulate the downstream signaling pathways, NF-κB and p-JNK pathways [41]. This increases the expression of inflammatory cytokines such as IL-1β and TNF-α, induces phosphorylation and degradation of IκBα, and accelerates the nuclear translocation of NF-κB [42]. In addition, activation of Caspase-1 induces inflammatory response through the pyroptosis mechanism, which further aggravates the damage to intestinal tissue [43]. Also, the expression of TJ proteins occludin and claudin-1 was significantly reduced in the PM exposure group, indicating a weakening of the bond between intestinal epithelial cells [24]. TJ proteins are key elements responsible for the physical protection of the intestinal barrier, and their loss leads to the leaky gut phenomenon and facilitates the penetration of external antigens, toxic substances, and pathogenic microorganisms into the intestinal tract [44]. As a result, this leads to hyperactivation of the intestinal mucosal immune system and persistent inflammatory responses, leading to failure to maintain intestinal tissue homeostasis [45]. However, EFEL effectively inhibited this pathological mechanism. EFEL restored the balance of intestinal microflora by promoting the growth of beneficial bacteria and inhibiting the proliferation of pathogenic strains. In particular, the recovery of *Lactobacillus*, *Alistipes*, and *Alloprevotella* plays an important role in alleviating the inflammatory environment by contributing to increased SCFA production, pH regulation, and intestinal mucosal immune regulation [46]. In addition, EFEL reduced the expression of IL-1β, TNF-α, and Caspase-1 by suppressing the TLR4/NF-κB/JNK inflammatory signaling pathway, restored the expression of occludin and claudin-1, and preserved the intestinal barrier function. The intestinal tissue protective effect of EFEL may be due to its various bioactive components. In particular, 5-*O*-caffeoylquinic acid has a strong antioxidant effect and relieves oxidative stress, and quercetin and its derivatives, such as quercetin-*O*-hexoside, and quercetin-*O*-acetylhexoside, are known to inhibit the NF-κB pathway and increase TJ protein expression [34, 47]. Moreover, rutin and luteolin-O-acetylhexoside can contribute to maintaining the function of the intestinal barrier by inhibiting the production of inflammatory cytokines and improving the intestinal inflammatory environment [35, 48]. The complex bioactivities of these components are thought to be closely related to the protective effects of EFEL, such as TJ protein recovery, inflammatory signal inhibition, and intestinal microbiota balance regulation, confirmed in this study.

PM_2.5_ is known to induce intestinal oxidative stress, inflammatory responses, and apoptosis of intestinal epithelial cells [49]. Heavy metals, organic compounds, and fat-soluble toxic substances contained in PM_2.5_ damage mitochondrial function in intestinal epithelial cells, induce excessive ROS production, and disrupt intracellular homeostasis [50]. This process activates the intrinsic apoptosis mechanism through the mitochondrial pathway, and the expression of the pro-apoptotic protein Bax increases while the anti-apoptotic protein Bcl-2 decreases [51]. This change in protein expression is shown by an increase in the Bax/Bcl-2 ratio, which indicates an increase in mitochondrial outer membrane permeability and activation of apoptosis-inducing signals [52]. This study confirmed an increase in Bax expression, a decrease in Bcl-2 expression, and a significant increase in the Bax/Bcl-2 ratio in the PM_2.5_ exposure group, indicating that the apoptosis pathway was activated in intestinal epithelial cells. This intestinal apoptosis is closely related to damage to the intestinal mucosa and deterioration of the intestinal barrier function, which may result in the aggravation of the intestinal inflammatory response and the collapse of immune homeostasis [53]. EFEL effectively inhibited this PM_2.5_-induced apoptotic response. When EFEL was treated, Bax expression decreased, Bcl-2 expression was restored, and the Bax/Bcl-2 ratio was also significantly reduced, demonstrating that EFEL contributed to the inhibition of apoptosis. This is due to the antioxidant and anti-inflammatory properties of chlorogenic acid, rutin, and quercetin derivatives contained in EFEL [14, 35]. These components are thought to promote the survival of intestinal epithelial cells through ROS removal, mitochondrial stabilization, and regulation of intracellular survival signals [36, 54].

To better understand the interaction between intestinal microbiota changes and host responses, correlation analysis was performed between major microbiota groups and physiological indicators in intestinal tissues. As a result of the analysis, pathogenic bacteria such as *Helicobacter* and *Clostridia* UCG-014 showed a significant positive correlation with inflammatory indicators such as TLR4, IL-1β, and TNF-α, as well as with apoptosis-related proteins such as BAX and Caspase-1. On the other hand, beneficial bacteria such as *Lactobacillus*, *Alistipes*, and *Muribaculaceae* showed a significant positive correlation with tight junction proteins, such as occludin and claudin-1, and BCl-2. These results suggest that the imbalance of intestinal microbiota is closely related to the key mechanism that induces inflammation and apoptosis of the intestinal mucosa, and that EFEL contributes to the maintenance of intestinal tissue homeostasis by simultaneously regulating multiple signaling pathways such as intestinal microbiota regulation as well as inflammation, intestinal barrier function, and apoptosis inhibition in the host.

## Conclusion

In summary, the EFEL suppressed the intestinal barrier disruption and microenvironmental dysregulation induced by PM_2.5_ exposure. The supplementation of EFEL restored the antioxidant defense system. It alleviated intestinal inflammation by regulating oxidative stress, TJ protein expression, and pro-inflammatory signaling pathways such as TLR4/NF-κB and JNK. Furthermore, EFEL improved gut microbial composition by increasing beneficial bacteria and reducing pathogenic bacterial overgrowth. These results suggest that EFEL might be a promising functional food material or a nutraceutical with a protective potential against PM_2.5_-induced intestinal dysfunction with the regulation of oxidative stress, inflammatory responses, gut microbiota, and epithelial barrier function. Despite these promising results, this study has several limitations that should be considered. Although the protective effects of EFEL against PM_2.5_-induced intestinal dysfunction were demonstrated, the mechanistic linkage between specific bioactive compounds and their direct interaction with intestinal cells or microbiota was not fully elucidated. Additionally, although alterations in gut microbiota composition were observed, functional analyses of microbial metabolites, such as bile acids and tryptophan metabolites, which would be valuable to strengthen the gut–microbiota–host interaction mechanisms, were not included. Although HT-29 cells are widely used as an *in vitro* model for human intestinal epithelial cells due to their goblet-like features and responsiveness to inflammatory stimuli, they are derived from colorectal adenocarcinoma and may not fully mimic normal epithelial physiology. Future studies using normal epithelial cells or human intestinal organoids will be necessary to validate these findings in a more physiologically relevant model.
